# Discovery and identification of a male-killing agent in the Japanese ladybird *Propylea japonica *(Coleoptera: Coccinellidae)

**DOI:** 10.1186/1471-2148-10-37

**Published:** 2010-02-11

**Authors:** Tamsin MO Majerus, Michael EN Majerus

**Affiliations:** 1 Institute of Genetics, University of Nottingham, Queen's Medical Centre, Nottingham, NG7 2UH, UK; 2 Department of Genetics, University of Cambridge, Downing Street, Cambridge, CB2 3EH, UK

## Abstract

**Background:**

Endosymbionts that manipulate the reproduction of their hosts have been reported widely in invertebrates. One such group of endosymbionts is the male-killers. To date all male-killers reported are bacterial in nature, but comprise a diverse group. Ladybirds have been described as a model system for the study of male-killing, which has been reported in multiple species from widespread geographic locations. Whilst criteria of low egg hatch-rate and female-biased progenic sex ratio have been used to identify female hosts of male-killers, variation in vertical transmission efficiency and host genetic factors may result in variation in these phenotypic indicators of male-killer presence. Molecular identification of bacteria and screening for bacterial presence provide us with a more accurate method than breeding data alone to link the presence of the bacteria to the male-killing phenotype. In addition, by identifying the bacteria responsible we may find evidence for horizontal transfer between endosymbiont hosts and can gain insight into the evolutionary origins of male-killing. Phylogenetic placement of male-killing bacteria will allow us to address the question of whether male-killing is a potential strategy for only some, or all, maternally inherited bacteria. Together, phenotypic and molecular characterisation of male-killers will allow a deeper insight into the interactions between host and endosymbiont, which ultimately may lead to an understanding of how male-killers identify and kill male-hosts.

**Results:**

A male-killer was detected in the Japanese coccinellid, *Propylea japonica *(Thunberg) a species not previously known to harbour male-killers. Families produced by female *P. japonica *showed significantly female-biased sex ratios. One female produced only daughters. This male-killer trait was maternally inherited and antibiotic treatment produced a full, heritable cure. Molecular analysis identified *Rickettsia *to be associated with the trait in this species of ladybird.

**Conclusion:**

We conclude that *P. japonica *is host to a bacterial male-killer that is vertically inherited with variable transmission efficiency. *Rickettsia *presence correlates with the male-killing trait, but there is some variation in the phenotypic expression of the trait due to interaction with host factors. Phylogenetic analysis using the 16S rRNA and 17 kDa antigen genes suggests there may have been horizontal transfer of *Rickettsial *male-killers between different ladybird hosts.

## Background

Invertebrate reproductive systems are frequently manipulated by inherited symbionts. In insects, three forms of manipulation, cytoplasmic incompatibility, feminisation of genetic males, and induction of parthenogenesis, are largely the province of a single genus of bacteria, the *Wolbachia*. However, a fourth manipulation, the biasing of secondary host sex ratios in favour of females by killing males, has been shown to have evolved in six different clades of bacteria: Group II *Spiroplasma *from *Drosophila *species, first identified in *Drosophila willistoni *[1]; Group VI *Spiroplasma *from *A. bipunctata *[2], *Harmonia axyridis *[3], *Danaus chrysippus *[4] and *Anisosticta novemdecimpunctata *[5]; γ-Proteobacteria, from *Nasonia vitripennis *[6] and *Cheilomenes sexmaculatus *[7]; *Rickettsia *from *Adalia bipunctata *[8] and *Adalia decempunctata *[9]; a Flavobacterium from *Colemegilla maculatus *[10], *Adonia variegata *[11] and *Coccinula sinensis *[7] and *Wolbachia *from *A. bipunctata*, several species of *Acraea *butterfly, first reported in *Acraea encedon *[12], another butterfly, *Hypolimnas bolina *[13] and two *Drosophila *species, *D. bifasciata *[14] and *D. innubila *[15]. The diversity of male-killers identified to date raises the question as to whether male-killing is a potential property of a few, many or almost all maternally inherited bacteria. To address this question, more bacteria associated with the male-killing phenotype need to be characterised and placed phylogenetically.

In addition, the vertical transmission efficiency of different male-killing bacteria varies from almost perfect (> 99% for *Spiroplasmas *in *A. bipunctata *and *H. axyridis *and one strain of *Wolbachia *in *A. bipunctata*) to less than 72% for *Rickettsia *in some samples of *A. bipunctata *[16-18]. The vertical transmission efficiency is a critical parameter in the dynamics of male-killer invasion and spread [19]. Assessments of the vertical transmission efficiencies of newly discovered male-killers are thus valuable to allow assessment of whether levels of vertical transmission are consistent within bacterial clades, or vary case by case as a result of interactions between each symbiont and its host.

The aphidophagous coccinellid beetles are a 'hot-spot' for invasion by male-killers [20]. Aspects of the ecology and certain life-history traits of these beetles mean that female progeny of infected females gain an advantage over those of uninfected females as a result of the death of male siblings, through the reallocation of resources from the dead males. Aphidophagous coccinellids lay eggs in batches, indulge in sibling egg cannibalism/consumption and suffer high neonate larval mortality due to starvation if they fail to catch and subdue aphid prey within a short period after dispersing from their egg clutch. Consequently, the daughters of females infected with a male-killer will benefit from the consumption of the soma of their dead (male-killed) brothers. The additional nutrients thus gained, which will be denied to daughters of uninfected females, reduce the starvation likelihood of daughters of infected females, for they have a longer time to search before starving to death. Furthermore, they are larger, so that they may be able to catch and subdue a greater size range of aphids [20].

Female-biased sex ratios, shown or assumed to result from male-killing have been reported in three species of Japanese coccinellid, *Harmonia axyridis *[17,21], *Cheilomenes *(= *Menochilus*) *sexmaculatus *[22] and *Coccinula sinensis *[23]. We here report the detection and identification of a male-killer in a fourth Japanese coccinellid, *Propylea japonica *(Thunberg). We show that the male-killer involved belongs to a bacterial clade known to be associated with male-killing, but not previously reported from Japan. Finally, we assess the male-killer's vertical transmission efficiency and compare this level with male-killers of the same clade from other coccinellid hosts.

## Results

### Detection of the male-killing trait

The sex ratio (proportion male) in a sample of *P. japonica *from Fuchu was 0.426 (26 male: 35 female). This does not differ significantly from 1:1 (Binomial exact test, p > 0.24).

The egg hatch rates of 13 *P. japonica *families (derived from the Fuchu sample above), all of which produced both male and female progeny, varied from 0.34 to 0.92 (Table 1). Ten matrilines, in which egg hatch rates were greater than 0.5 and progenic sex ratios were not significantly different from 1:1, were designated normal (N). Three families (PF2, PF3, PF5), with hatch rates below 0.5 produced significantly female biased sex ratios, but with some males, and were designated female biased (FB).

**Table 1:** The egg hatch rates and progenic sex ratios of *P. japonica* matrilines, from Fuchu.

Line	Egg hatch rate	N° of progeny	Sex ratio	Status
PF1	0.613	36	0.528	N
PF2	0.434	17	0.235	FB
PF3	0.338	34	0.235	FB
PF4	0.657	45	0.467	N
PF5	0.395	20	0.250	FB
PF7	0.528	32	0.375	N
PF8	0.764	66	0.561	N
PF9	0.924	78	0.560	N
PF10	0.870	67	0.493	N
PF12	0.462	49	0.000	SR
PF13	0.678	53	0.472	N
PF14	0.778	84	0.488	N
PF15	0.673	59	0.576	N
PF16	0.764	90	0.467	N

One matriline (PF12) produced 49 female progeny and no males. The egg hatch rate from this female was 0.462. This low hatch rate is consistent with male-killing, and in conjunction with the total absence of males leads to the conclusion that line PF12 bears a male-killing element, designated sex ratio (SR).

**Figure 1:**
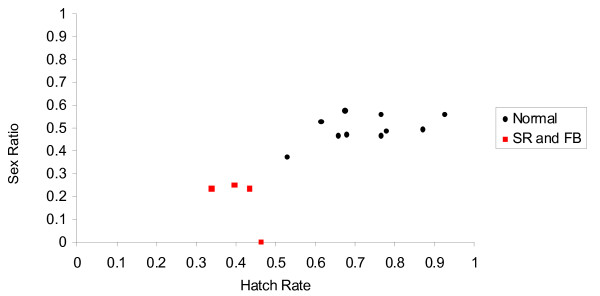
**Egg hatch rate versus sex ratio for N and SR/FB matrilines of Fuchu *P. japonica***.

Egg hatch rates of the SR and three FB lines were all below 0.462, while those of the N lines all exceeded 0.528. The difference between the two groups was significant (χ^2 ^= 155.954, d.f. = 1, p < 0.001). The relationship between egg hatch rate and progenic sex ratio is illustrated in Figure 1. The prevalence of the trait in this sample depends crucially upon the true infection status of the three FB lines. Additional phenotypic and molecular analysis was carried out on these lines and is discussed below.

### Inheritance and vertical transmission of the male-killing trait

#### SR Line

In crosses designed to examine the inheritance of the male-killing trait in the SR line PF12, two of the pairs (PF12.1, PF12.9) failed to produce sufficient adult progeny for analysis. Results from the remaining eight pairings are given in Table 2. All the progeny of these eight pairs produced over this period were female (n = 109), with the egg hatch rates being less than 0.5 for each pair. The SR status in these lines is maternally inherited.

**Table 2:** The inheritance of the SR trait in *P. japonica*.

Line	Number of eggs	Egg hatch rate	Number of progeny	Sex ratio (proportion male)	Status
PF12.2	165	0.285	20	0	SR
PF12.3	91	0.275	17	0	SR
PF12.4	89	0.202	10	0	SR
PF12.5	46	0.413	13	0	SR
PF12.6	117	0.248	14	0	SR
PF12.7	79	0.266	13	0	SR
PF12.8	70	0.229	11	0	SR
PF12.10	120	0.225	11	0	SR

Data from initial crosses of PF12 F_1 _females, prior to treatments with tetracycline.

#### FB and N Lines

Crosses involving N F_1 _females all had high hatch rates (>0.6) and produced progenic sex ratios that did not differ significantly from 1:1 (n > 16 in all cases), irrespective of whether males were drawn from N families, FB families, or from F_2 _progeny of the SR family, PF12, after treatment with tetracycline. Further, there was no evidence of a female biased sex ratio in the F_2 _of any of the N families tested.

It was found that the FB lines all had high hatch rates and produced roughly equal numbers of male and female progeny (data available on request), suggesting that the female bias in these lines may not be due to the presence of a male-killer. This result was assessed further by molecular analysis.

It is concluded that the female bias in PF12 is the result of distortion of the secondary sex ratio by a male-killer. Furthermore, the SR trait in PF12 is maternally inherited with a very high vertical transmission rate.

### Assessment of vertical transmission efficiency

The vertical transmission efficiency of the SR trait from mother to adult daughter, calculated as 1 - (the number of males produced divided by the number of females produced) [24], assessed from PF12, PF12.2-4, PF12.7, PF12.8, PF12.10 and the ten additional PF12 F_1 _pairs was 0.998 (2 males in 878 progeny).

### Sensitivity to tetracycline

The results of experiments to assess the effect of tetracycline treatment are given in Table 3. (Table 2 shows the egg hatch rates and progenic sex ratios for these pairs prior to these treatments.) The egg hatch rates and progenic sex ratios for the pairs administered tetracycline increased following this treatment, indicating that the antibiotic produces alleviation of the effects of the male-killer, as would be expected if it is bacterial. There was no significant change in either trait in the four control pairs.

**Table 3:** Effect of tetracycline treatment of PF12 F_1 _females.

Pair	Egg hatch rate	Number of progeny	Sex ratio (proportion male)	Status
Control 1, no alternative food				
PF12.3	0.215	14	0	SR
PF12.10	0.316	22	0	SR

Control 2, golden syrup				
PF12.4	0.395	35	0	SR
PF12.8	0.266	22	0	SR

Tetracycline treatment				
PF12.5	0.446	24	0.458	Cured
PF12.6	0.602	36	0.556	Cured

Pre-treatment egg hatch rates and sex ratios are given in Table 2.

### Does tetracycline treatment produce a heritable cure of the SR trait?

As there was no effect of the control treatments on PF12.3, PF12.4, PF12.8 and PF12.10, it is not surprising that all eight pairs from these lines retained their SR status (egg hatch rates < 0.5 and only female progeny) in the next generation. Conversely, all four females from the two tetracycline treated lines produced increased egg hatch rates and progenic sex ratios that did not differ significantly from 1:1 (Table 4).

**Table 4:** The inheritance of the antibiotic cure of the SR trait in *P. japonica*.

Pair	Egg hatch rate	Number of progeny	Sex ratio (proportion male)	Status
PF 12.5.i	0.67	23	0.609	N
PF12.5.ii	0.88	34	0.412	N
PF12.6.i	0.92	56	0.446	N
PF12.6.ii	0.77	18	0.556	N

Results of breeding from female progeny of tetracycline treated lines.

It is concluded that treatment with tetracycline produces a full heritable cure of the SR trait, with an increase in egg hatch rate and concomitant production of male-progeny. The effect of tetracycline treatment occurred within four days of the commencement of treatment. These factors strongly suggest that the male-killer is bacterial in nature.

### Identification of the agent associated with male-killing

To determine the identity of the causal agent of male-killing in PF12, on the assumption that the male-killer was a bacterium, the 16S rRNA gene was characterised from two F_1 _females from PF12.

BLAST database comparison of the 16S rDNA sequence isolated from two PF12 F_1 _females listed *Rickettsia bellii *as the closest match (99% identity) [EMBL: FN550103]. It is concluded that the SR trait in *P. japonica *is caused by a male-killing α-proteobacterium of the genus *Rickettsia*.

### Correlation between the SR trait and Rickettsia presence

In order to ascertain whether *Rickettsia *presence correlates with the male-killing phenotype a larger number of individuals were tested. Females from the parental, F_1 _and F_2 _generations of the SR line, PF12, individuals from each of the three FB lines, along with one female and one male from each of seven of the N lines, three female progeny from PF12.8 after golden syrup treatment and two male and two female progeny from each of PF12.5 and PF12.6 produced after tetracycline treatment, were screened via a *Rickettsia*-specific PCR from the 17 kDa antigen gene. In addition, the parental female of one line (PF11) and an F_1 _female of another (PF6) that had failed to produce enough progeny for analysis, were tested in the same way.

None of the individuals from N lines gave a positive result with *Rickettsia*-specific primers, confirming that absence of the bacteria coincides with absence of the male-killing trait. Conversely, all the individuals from the PF12 matriline, gave a positive 17 kDa antigen gene PCR result [EMBL:FN550104], with the exception of one female, produced in the control line PF12.8 from the antibiotic sensitivity experiment. All the other individuals from the antibiotic sensitivity experiment (post-treatment) tested positive for *Rickettsia*. This confirms that the bacteria in line PF12 are vertically inherited with very high transmission efficiency. Furthermore, it suggests that where alleviation of the symptoms of male-killing has occurred following antibiotic treatment, bacteria (or bacterial DNA) may still be present.

The situation in the FB individuals is less clear cut. For PF3, six F_1 _females were tested, one of which was positive. For PF5, seven F_1 _females were tested, three of which were positive. Although larger numbers of individuals would be required to be confident that this was an accurate indicator, these results suggest very low vertical transmission efficiency in these two lines. This could be due to a very low bacterial density in the parental female, meaning that only a small proportion of her eggs inherited the infection. Alternatively, there may be some host interaction with the bacteria that reduces their transmission to the eggs. For PF2, the parental female tested negative, suggesting that the female bias in this line may have another cause and can be considered to represent a type one error.

The two female individuals (PF6 and PF11) lacking breeding data that were tested did produce a PCR product band. This indicates that they were infected with *Rickettsia *and it may be suggested that if they had produced sufficient progeny for analysis they would have exhibited the phenotypic indicators of male-killing.

Combining the phenotypic data with the molecular identification of *Rickettsia *infection in the two incompletely ascertained lines brings the prevalence of the male-killing trait in this sample of *P. japonica *to 0.3125 (5 out of 16).

It is concluded that where it has been possible to test, *Rickettsia *presence correlates with the SR trait, but that there is some variation in the phenotypic expression of the trait, either due to interaction with host factor(s), low bacterial density or variable bacterial vertical transmission efficiency.

### Phylogenetic analysis

To assess the phylogenetic position of the *Rickettsia *male-killer from *P. japonica *two phylogenetic trees were produced. Sequences chosen for comparison comprised those most similar as identified by a BLAST search [25] and other relevant related bacteria, with known reproductive phenotypic effects. Where possible the same species were included in both trees to allow comparisons between the two to be made, however data were not always available to allow this.

**Figure 2:**
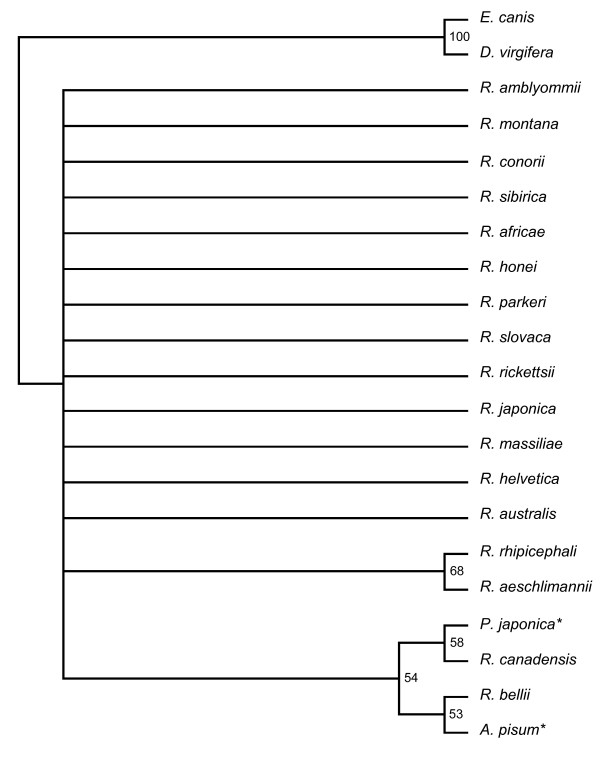
**16S rDNA phylogenetic tree indicating the position of the *P. japonica *male-killer amongst the *Rickettsias***. * indicates endosymbiont host name; *mk *indicates male-killer.

**Figure 3:**
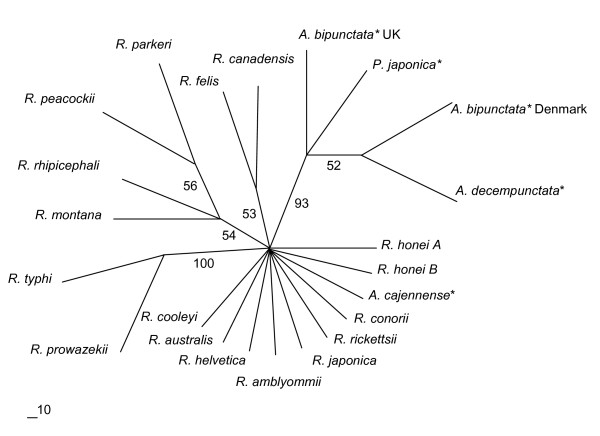
**17 kDa antigen gene phylogenetic tree indicating the position of the *P. japonica *male-killer amongst the *Rickettsias***. * indicates endosymbiont host name; *mk *indicates male-killer.

The 16s rDNA sequence from the *P. japonica *male-killer was aligned with those from 18 other *Rickettsias *and two outgroups (*Wolbachia *and *Erlichia*) (Figure 2). This tree is not particularly informative. The *P. japonica *male-killer appears most closely related to *R. canadensis*, followed by *R. bellii *and, interestingly, a secondary endosymbiont of *A. pisum*. However, the bootstrap values on this tree, with the exception of that separating the outgroups from the rest, are all fairly low. Also full 16S rDNA sequences for the *A. bipunctata *and *A. decempunctata *male-killers were not available meaning this tree can not be used for phylogenetic comparison with other ladybird male-killers. Hence, a second tree was produced, aligning the 17 kDa antigen gene sequences from the *P. japonica *male-killer and those from 21 other *Rickettsia *species, including the male-killers from *A. bipunctata *and *A. decempunctata *(Figure 3).

This tree is broadly consistent with the 16S tree, although again many of the bootstrap values are low. The ladybird male-killers form a clade distinct from the other *Rickettsias*. The *P. japonica *male-killer is most closely related to that from *A. bipunctata *from the U.K., whereas the male-killer from *A. bipunctata *from Denmark clusters with that from *A. decempunctata*. This is suggestive of horizontal transfer between ladybird species (see below for further discussion).

## Discussion

The results of these studies have revealed sex ratio distortion in a single matriline of *P. japonica*, leading to severely female biased sex ratios in this line over three generations. The trait is maternally inherited, is sensitive to tetracycline treatment and shows a strong correlation with presence of a *Rickettsia*. In addition, the same bacteria appear to produce a much weaker male-killing phenotype in two other matrilines, leading to less strongly, but still significantly, female-biased sex ratios in these lines.

The phylogenetic analysis of this *Rickettsia *male-killer confirms that it is closely related to the other male-killers in the analysis. Further, the 17 kDa antigen tree provides some evidence of horizontal transfer between ladybird hosts, as the male-killers in the two populations of *A. bipunctata *are more closely related to those in *P. japonica *and *A. decempunctata*, than they are to each other. Whilst there has been no demonstration of how ladybirds might acquire male-killers in the wild, it is tantalizing to note that the aphid prey species *A. pisum *contains bacteria phylogenetically related to those causing male-killing in the three ladybird species. Further molecular investigation of these bacteria, involving more rapidly evolving regions of the genome may allow further insight into possible origins of bacterial male-killers.

As similar members of bacterial clades are found to cause male-killing in related hosts, such as a single family of beetles, comparisons of the vertical transmission efficiencies of the male-killers can be made both within and between bacterial clades. In this study, the third instance of male-killing within the Coccinellidae has been found for *Rickettsia*. Flavobacteria and *Spiroplasma *have been identified in 3 and 2 instances respectively (see Table 5). In addition, it has previously been established that male-killing is caused by two different strains of *Wolbachia *in a single species of coccinellid, *A. bipunctata *[16].

**Table 5:** Summary of the vertical transmission efficiencies of male-killing bacteria in different coccinellid hosts.

Male-killer	Host	Vertical transmission efficiency	Reference
*Rickettsia*	*Adalia bipunctata*	0.85, Bielefeld; 0.72, Moscow; 0.87, Cambridge (mean value)	[16] [24]
*Rickettsia*	*Adalia decempunctata*	>0.9	[9]
*Rickettsia*	*Propylea japonica*	0.998	This study
Flavobacterium	*Coleomegilla maculata*	Variable, progressive 0.46-1.00	[35]
Flavobacterium	*Adonia variegata*	>0.99	[11]
Flavobacterium	*Coccinula sinensis*	0.991	[7,23]
*Spiroplasma*	*Adalia bipunctata*	1.00, Bielefeld; 0.99, Moscow; 1.00, Tuva	[16]
*Spiroplasma*	*Harmonia axyridis*	>0.99, Sapporo	[17]
*Wolbachia *Y	*Adalia bipunctata*	0.99, Moscow;	[16]
*Wolbachia *Z	*Adalia bipuncatata*	0.86, Moscow, 1.00, Tomsk	[16]

Examination of the vertical transmission efficiencies of the bacterial male-killers in each of these four clades (Table 5) shows transmission efficiencies to be highly variable both between clades and in most cases within clades.

Variation in vertical transmission is most clearly demonstrated by the *Rickettsial *male-killers. For some lines of *P. japonica *the vertical transmission efficiency appeared to be very low (< 50%), based on assessment of male-killer status via molecular analysis, whilst in PF12 it appeared to be close to 100%. The *A. decempunctata *male-killer exhibits vertical transmission efficiency greater than 90%, at least for some lines, whereas *Rickettsia *infected lines of *A. bipunctata *consistently have lower vertical transmission efficiencies of between 72% and 87%. Again there is evidence of inter-individual variation in different matrilines of *A. bipunctata*, with lines exhibiting vertical transmission efficiencies as low as 54% [18].

The male-killing Flavobacterium in *C. maculata *shows highly variable vertical transmission, both between families and within the lifetime of single females that exhibit a progressive sex ratio trait. This differs from the vertical transmission efficiency of the *A. variegata *male-killer, which is generally high, but can exhibit progressive sex ratio (Majerus, M. pers. comm.) and that of the *C. sinensis *male-killer which is high, with few males being produced by infected females.

Whilst caution must be exercised when considering single lines, particularly when the number of individuals may be small, it is pertinent to note that variation in either the host or endosymbiont genotype is likely to play a part in these differences. Although it is not possible to rule out environmental factors affecting the phenotypic expression of the male-killer, it is unlikely that these can be the main causes of the differences seen here. This is due to the fact that the breeding experiments for all six of these host species were carried out in the same laboratory under fairly constant conditions.

The correlation between *Rickettsia *presence and the SR trait in this matriline, ascertained using the 17 kDa gene was exact, apart from in progeny of tetracycline treated females. Here, females 'cured' of the SR trait and their male siblings gave positive results when assayed for *Rickettsia*. There are two possible explanations of the apparent presence of *Rickettsia *in non-killed males and 'cured' females, despite the evidence that tetracycline produces a full heritable cure of the trait. First, bacteria may be present in very low numbers in the progeny of treated individuals. If the pathogenicity of the *Rickettsia *towards males is density dependent, as has been suggested e.g. for the *Rickettsia *male-killer of *Adalia decempunctata *[9], there will be alleviation of male-killing expression, despite continued bacterial presence. Tetracycline is bacteriostatic, hence time is required for the bacteria to be eliminated by the host. Some bacteria may pass into the egg and hence into the progeny, despite having their growth inhibited by the antibiotic. As PCR is a very sensitive test, a very small number of target molecules in the DNA input would be enough to generate a visible product. Second, the apparent presence of *Rickettsia*, may be the result of PCR amplification of DNA from dead bacteria. Bacteria may pass into the egg before death and DNA from dead bacteria may be amplified. The alleviation of male-killing symptoms would then be due to a lack of viable bacteria and not necessarily dependent on bacterial density.

A model in which a threshold bacterial density is required to cause male-killing can similarly explain the situation in the 2 FB lines for which a proportion of the female progeny tested positive for *Rickettsia *presence, but which were part of sibships where males survived. If it is assumed that the maternal bacterial levels were low, survival of male embryos and hence non-SR phenotypic status would follow. However, it could be argued that in this instance increase in bacterial numbers should lead either to late male-killing or progressive sex ratio once enough time has elapsed and potentially that once a high enough bacterial density had been established in the parental female, high vertical transmission (as shown by PF12) would be exhibited by all infected females. In this sample of *P. japonica*, however, virtually none of the unhatched eggs were grey. This suggests that, at least during egg development, male-killing occurs very early and there is no further male-death later in development after bacterial multiplication. This supports the suggestion that host factors are affecting the actions of the male-killer; however, it would fit equally well with a model in which the endosymbiont either kills early or not at all. Interestingly, male-killing *Wolbachia *in *D. innubila *show variation in transmission and expression, and this variation is correlated with the density of *Wolbachia *within host flies [26] and has been shown not to be related to host genetic factors [27]. It would be worthwhile investigating both male-specific effects on egg hatch-rate and progenic sex ratio, as well as screening larger numbers of males and females for *Rickettsia *presence and testing for variation in bacterial density in FB and SR families of this species.

## Conclusion

*Propylea japonica *is host to a *Rickettsia *male-killer that is closely related to *Rickettsia *male-killers in two other ladybird host species. Phylogenetic analysis indicates that there may have been horizontal transmission of *Rickettsia *within the clade of ladybird male-killers. There is wide variation in vertical transmission efficiency of the *Rickettsia *male-killers, both within and between the different host species, indicating that host genetic variation may be influencing the phenotypic expression of these male-killers.

## Methods

### Detection of the male-killing trait

A sample of 61 *P. japonica *was collected by eye from the campus of Tokyo University of Agriculture and Technology, Fuchu, Tokyo, Japan, between 25th August 1998 and 2nd September 1998 as part of a survey of 30 coccinellid species for male-killers [7]. The sample was kept in a domestic refrigerator until 4th September 1998, when it was transported to Cambridge, England. In Cambridge the beetles were transferred to Petri dishes, kept at 21°C and fed on *Acyrthosiphon pisum *(Harris). The sample was sexed, by examination of the posterior abdominal sturnites, under CO_2 _anaesthetic. Fourteen mating pairs were removed from stock dishes to establish individual matrilines. Eggs laid by these females were collected and counted over a two-week period. Once eggs had hatched and larvae had dispersed from egg clutches, egg hatch rates were assessed, egg remnants being classed as clear (hatched normally), grey (fertile but inviable for unknown reason including possibly male-killed) or yellow (infertile or male-killed) [18]. Larvae were fed daily on *A. pisum *and allowed to pupate and eclose in the dishes. Progenic sex ratios were recorded.

### Nomenclature

The matrilines from the original sample were designated PF (**P**ropylea from **F**uchu) and then a number (1-14). Subsequent generations show the parental name followed by a '.' to indicate a new generation, and then a number e.g. PF12.3 is the third cross generated from F_1 _female progeny of PF12 and PF12.5.i is the first cross generated from an F_2 _female, progeny of PF12.5.

### Inheritance and vertical transmission of the male-killing trait

#### SR Line

Ten virgin females from the SR matriline, PF12, were mated with males from N matrilines. Eggs were collected over a one week period and progeny reared as for the initial matrilines. Egg hatch rates and progenic sex ratios were recorded.

#### FB and N Lines

The three FB lines and four N lines were further analysed to look at the inheritance of the trait. Four virgin females from each of the FB families PF2, PF3 and PF5, and one from each of four N families were mated with males from N matrilines. One virgin male from each of the FB families was mated to a virgin female from N families. Further, two F_2 _males from F_1 _families (PF12.5, PF12.6) from the SR matriline which produced male progeny only subsequent to treatment with tetracycline (see below) were mated to unrelated females from N lines. Again, these families were treated as before, egg hatch rates and progenic sex ratios being recorded.

### Assessment of vertical transmission efficiency

The vertical transmission efficiency of the SR trait was assessed on the basis of the proportion of male progeny in the PF12 matriline. To increase the stringency of assessment of this vertical transmission estimate, ten F_1 _females from the SR line were mated to unrelated N males. Progeny were reared as before and the progenic sex ratios were recorded.

### Sensitivity to tetracycline

To determine the nature of the male-killing element, six of the PF12 F_1 _pairs that had produced all female progeny were selected for tests to determine whether the sex ratio trait was affected by antibiotic treatment. Two of these females (PF12.5, PF12.6) were fed a 10% solution of tetracycline in golden syrup daily for an hour. Two further females (PF12.4, PF12.8) were offered just golden syrup daily for an hour. The final two females (PF12.3, PF12.10) were not fed anything other than their normal *A. pisum *diet. Assessment of egg hatch rates and progenic sex ratios continued subsequent to these treatments.

### Does tetracycline treatment produce a heritable cure of the SR trait?

Two female progeny of each of the two lines treated with tetracycline, from eggs laid 12 days after commencement with treatment were mated to males from N families. Eggs were collected, progeny reared and egg hatch rates and progenic sex ratios recorded as previously. Two female progeny from each of the four control lines were treated in the same way.

### Identification of the agent associated with male-killing

Genomic DNA was extracted from whole ladybirds killed in liquid nitrogen and ground up. Samples were incubated in 250 μl of DNA extraction buffer (80 mM EDTA, 1% SDS, 160 mM sucrose, 100 mM Tris HCl pH 8.0), containing 20 μg proteinase K, at 37°C, overnight. An equal volume of 25:24:1 phenol:chloroform:isoamyl alcohol was added, the tubes shaken thoroughly and then spun at 13,000 rpm for 10 minutes. The aqueous layer was removed to a new tube and the previous step repeated with an equal volume of 24:1 chloroform: isoamyl alcohol. The DNA was precipitated using 1/2 volume of 7.5 M NH_4_-AC and 2 volumes of 100% ethanol, washed with 70% ethanol and resuspended in 100 μl of sterile distilled water.

Each DNA sample was assayed for the presence of bacteria by PCR using general bacterial 16S rDNA primers described by [28]. Primers 27f (5' - GAGAGTTTGATCCTGGCTCAG - 3') and 1495r (5' - CTACGGCTACCTTGTTACGA - 3') were used in 25 μl reactions containing 1 μl of the genomic ladybird DNA preparation; 2.5 μl NH_4 _buffer; 2.5 μl dNTPs (2 mM with respect to each dNTP); 1.25 μl MgCl_2 _(50 mM); 0.25 μl each primer (50 pmol/μl); 0.25 μl Taq polymerase and sterile distilled water to give a final volume of 25 μl. Amplification controls were run for the PCR cocktail and for an *A. bipunctata *female known to be infected with a male-killing *Rickettsia*. Cycle conditions on a Hybaid omnigene PCR machine were: 1 cycle of 2 min 94°C; 35 cycles of 15 s 94°C, 30 s 55°C, 3 min 72°C; 1 cycle 20 min 72°C. PCR products were visualised using UV light following electrophoresis on a 1% agarose gel containing ethidium bromide.

To determine the identity of the agent in those samples producing a positive 16S PCR result, the product band was purified from the agarose gel using Prep-a-Gene (Biorad) and directly sequenced using dye-labelled terminators in a cycle-sequencing reaction, the products being visualised on an ABI 373 automated sequencer. Both strands of the whole unit were sequenced using internal primers.

Control tests, with primers which amplify the COI gene from insect mtDNA [29], were performed on samples which failed to yield a product with the general bacterial 16S rDNA PCR, to check that the DNA preparation did not contain factor(s) inhibiting the PCR reaction.

### Correlation between the SR trait and Rickettsia presence

The 17 kDa antigen gene (primers R1, 5' - GCTCTTGCAACTTCTATGTT - 3' and R2, 5' - CATTGTTCGTCAGGTTGGCG - 3', [30]) was used to screen specifically for *Rickettsia*. Cycle conditions on a Hybaid omnigene PCR machine were: 1 cycle of 2 min 94°C; 35 cycles of 15 s 94°C, 1 min 60°C, 1.5 min 72°C; 1 cycle 10 min 72°C. For some individuals, the 17 kDa antigen gene PCR product obtained was sequenced for phylogenetic analysis (see below).

### Phylogenetic analysis

Sequences were aligned using the programme CLUSTALW [31]. Data sets were characterised by calculating the number and percentage of nucleotide differences between pairwise compared sequences. Fifty-six different substitution models were considered using the programme Modeltest (version 3.06) and the simplest model, for which the log likelihood could not be significantly improved by choosing a more complex alternative, was selected. Phylogenetic trees were reconstructed using an iterative search strategy, following Schulenburg *et al. *[32] using the program PAUP* version 4.0b8 [33]. An initial tree topology was obtained using unweighted maximum parsimony (MP) and a heuristic tree search via branch-swapping by tree bisection and reconnection. This served to obtain maximum likelihood (ML) estimates required for the selected substitution model (t_s_/t_v _ratio, values for the rates of six different types of substitutions, four distinct gamma rate heterogeneity categories and fraction of invariable sites). These parameters were then used for ML tree estimation. Here, the MP topology served as a starting tree for a heuristic search by nearest neighbour interchange. The robustness of the inferred tree topology was determined by non-parametric bootstrapping [34], with 100 replicates.

## Authors' contributions

TMOM carried out the majority of the breeding work, all the molecular and phylogenetic analysis and wrote the manuscript. MENM collected the original *P. japonica *sample, advised on study design and analysis, assisted with ladybird culturing and contributed to an earlier draft of the manuscript.
